# “If you don't find anything, you can't eat” – Mining livelihoods and income, gender roles, and food choices in northern Guinea

**DOI:** 10.1016/j.resourpol.2020.101939

**Published:** 2021-03

**Authors:** Ronald Stokes-Walters, Mohammed Lamine Fofana, Joseph Lamilé Songbono, Alpha Oumar Barry, Sadio Diallo, Stella Nordhagen, Laetitia X. Zhang, Rolf D. Klemm, Peter J. Winch

**Affiliations:** a Department of International Health, Johns Hopkins Bloomberg School of Public Health – 615 N Wolfe St, Baltimore, MD, 21205, USA; b Helen Keller International – One Dag Hammarskjold Plaza, Floor 2, New York, NY, 10017, United States; c Julius Nyerere University of Kankan, Kankan, Guinea; d Action Against Hunger USA, One Whitehall St, Second Floor, New York NY, 10004, United States; e Global Alliance for Improved Nutrition (GAIN), Rue de Vermont 37-39, 1202, Geneva, Switzerland

**Keywords:** Artisanal mining, Food choice, Women's workload, Income instability, Guinea

## Abstract

Artisanal and small-scale mining (ASM) continues to grow as a viable economic activity in sub-Saharan Africa. The health and environmental impacts of the industry, notably linked to the use of potentially toxic chemicals, has been well documented. What has not been explored to the same extent is how pressures associated with ASM affect food choices of individuals and families living in mining camps. This paper presents research conducted in 18 mining sites in northern Guinea exploring food choices and the various factors affecting food decision-making practices. Two of the most influential factors to emerge from this study are income variability and gender roles. Results from this study suggest that through artisanal mining, women have the opportunity to earn a larger income that would otherwise be unavailable through agriculture. However, this benefit of potentially earning a larger income is often reduced or constrained by existing gender roles both at the mines and in the home, such as disparity in pay between men and women and increased pressures on women's time. This limits the potential benefit to household food decision-making that could have been gained from higher income. These results do not seek to establish one livelihood as superior; rather, they demonstrate that even when presented with opportunities to earn higher incomes, women still face many of the same barriers and challenges that they would in other economic activities. Additionally, while work and time demands on women change upon arrival in the mining camps, existing gender roles and expectations do not, further restricting women's decision-making capacity.

## Highlights

• Artisanal mining brings greater earnings that miners can use to acquire food.
• Time constraints linked to mining, as well as domestic tasks, limit food choice.
• Unequal payment for mining work further restrict women's food choices.
• Income instability significantly impacts caregiver food decision-making abilities.

## 1 Introduction

An estimated 20–40 (IGF, 2017, USAID, 2020, World Bank, 2019)million individuals are engaged in the artisanal and small-scale mining (ASM) industry globally, with approximately 100 million additional people dependent on the incomes earned in the sector (Hilson et al., 2017). Approximately 10 million artisanal miners were estimated to be active in ASM in Sub-Saharan Africa (SSA) in 2019 (World Bank, 2019). While ASM has historically been viewed as populated by entrepreneurial individuals looking to “get rich quick”, the last two decades have seen a growing understanding of ASM as an established alternative source of revenue for the rural poor (Hilson et al., 2017; Hilson, 2012, 2016; Hilson and Garforth, 2012; Siegel and Veiga, 2009; Fahy Bryceson and Jønsson, 2009; Bryceson and Fahy Bryceson, 1996; Kamlongera, 2011). This paper contributes to this knowledge by adding a relatively unexplored dimension to the conversation: how mining conditions, income derived from mining, and traditional gender roles influence food decision-making in these communities.

Multiple economic and social factors have contributed to the sustained growth of ASM in sub-Saharan Africa (SSA), particularly in rural communities. Historically, rural poverty reduction strategies have focused on agriculture, aiming to increase the income of small-holder farmers through farm subsidies and interventions (Hilson, 2016; Kamlongera, 2011). Beginning in the 1990s, scholars and policymakers began to investigate income diversification strategies used by rural African households and recognized the significant role played by ASM, despite the lack of attention that had been paid to it as a viable source of income (Hilson and Garforth, 2012). The view that African farmers were self-sufficient, subsistence-based producers had been recognized as inaccurate, yet many livelihood programs continue to view activities such as ASM as something to supplement, rather than replace, farming (Bryceson and Fahy Bryceson, 1996). More recently, a growing body of literature views agricultural and ASM as complementary livelihoods (Hilson and Garforth, 2012; Cartier and Burge, 2011). For farmers with few alternatives, mining is an appealing alternative. Initial capital investment in artisanal and small-scale gold mining is minimal, and while labor-intensive, presents relatively few barriers to entry (Bryceson and Lahiri-Dutt, 2018; Stocklin-Weinberg et al., 2019). A growing body of work views the two industries as ones that coexist and are used by the rural poor simultaneously to diversify their income and build resilience to external shocks (Hilson, 2016; Kamlongera, 2011; Pijpers, 2014).

While the environmental and health impacts of ASM have been discussed extensively (World Health Organization, 2016 , other potential consequences of ASM remain largely unexamined, such as its impact on household food choices. Only a limited number of studies have examined issues related to food and nutrition in ASM communities. A qualitative study of mining camps in Northern Ghana found limited consumption of fruits and vegetables and high consumption of grains and oils by women (Long et al., 2015a), while a geographic mapping study in the Democratic Republic of the Congo found higher rates of malnutrition in areas with an elevated mining presence (Kandala et al., 2011). A recently published analysis of industrial mining and rural food security suggests that industrial mining operations decrease food availability among women, and hypothesizes that women's traditional roles in rural communities of cultivating subsistence crops makes them more vulnerable to food insecurity (Wegenast and Beck, 2020). While the analysis focuses on industrial mining activities, many of the mechanisms through which women may be vulnerable to food insecurity due to industrial mining (devaluing of traditional gendered roles for women in mining, competing pressures for time between a woman's domestic and livelihood responsibilities) can also be associated with ASM. In many rural sub-Saharan African contexts, lower income households spend large portions of their income on food purchases (Tangtrakul, 2010), in some settings up to 80% of a household's income (Bashir and Schilizzi, 2013). Traditional agricultural households routinely supplement household food supplies with self-production (Turner et al., 2018), however the extent to which informal mining households can supplement existing food gaps through self-production may be limited, given competing pressures on time and resources.

Gender also plays a significant role in determining a household's overall income and livelihoods. Women traditionally face significant challenges within the agricultural sector, and those challenges are replicated within the mining sector. Women make up an estimated 20–30% of the global ASM workforce (Hinton et al., 2006; Weldegiorgis et al., 2018), and in sub-Saharan Africa their participation in the sector is estimated to be even higher, at 40–50% of the total workforce (IGF, 2017; Hinton et al., 2006; Guédé et al., 2019; Huggins et al., 2017). The attention that has been paid to gender has often been through the lens of encouraging women to leave a sector seen by policymakers as having a high risk of economic and sexual exploitation (Huggins et al., 2017). This lack of attention contributes to an environment in which women's work is viewed informally, is unrecognized, or is viewed as supportive of, but not essential to, the main work of mining conducted by men (Buss et al., 2019). In practice, this excludes women from the most lucrative mining opportunities and prevents them from gaining additional skills or productive networks to increase future earnings (Guédé et al., 2019; Buss et al., 2019; Reichel, 2019). Traditionally, women's role in ASGM is a supportive one – i.e., they perform all tasks that support the primary focus of digging shafts and extracting gold. Such responsibilities include removing material that has been dug out from mining shafts, washing and sorting of material to separate gold from other materials, and transporting of material from the mining site to other locations for further processing (Huggins et al., 2017; Reichel, 2019; Veiga et al., 2006).

This paper presents results from a study conducted in Northern Guinea, where artisanal and small-scale gold mining is a key income-generating activity. The study sought to better understand the factors that constrain and motivate food choices of female miners. Results presented in this study demonstrate the powerful influence of existing gender norms surrounding women's work and the manner in which income is earned through mining on food choices. Influenced by these pressures, women often choose food for their family based on ease of access, available resources (both financial and temporal), and familiarity and comfort surrounding certain types of food.

## 2 Methodology

Research was conducted in northeastern Guinea, Kankan Region, Kouroussa and Siguiri prefectures. Study sites were selected purposively to cover four different strata of sites: temporary mining camps, villages far from larger towns, villages close to larger towns, and towns. Kouroussa and Siguiri prefectures were chosen as the site of the research for several reasons. The region has been a center of artisanal gold mining activity since at least the 12th century, is home to hundreds of thousands of artisanal gold miners, and produces several tons of gold annually (Veiga et al., 2006). A total of 18 sites were included, with samples from each strata of mining camp. Stratification and selection of sites was driven by the expectation that proximity to larger towns influences food choices. Before the cross-sectional survey and in-depth interviews were conducted, an initial scoping mission in May 2018 was conducted in Kouroussa and Siguiri prefectures to assess potential study sites, meet with local authorities, and gather preliminary data. During these initial interviews, lists of potential sites were provided by local authorities to cover four different strata of mining camps (with the strata chosen based on factors likely to affect access to food sources) – sites connected to central towns; sites connected to villages, near central towns; sites connected to villages, far from central sites; and sites connected to mining camps. This list was then narrowed to include only those sites known to be active during the rainy season, leaving 21 sites. This list was then subsequently divided into four groups based on the abovementioned strata. Eight sites were then selected for the research, stratified by stratum and district. One to three sites from each stratum were chosen, proportional to the total number of similar sites in a stratum, and divided by district. Final selection was based on random number selection, which was also used to generate backup lists of study sites, of which only one was used (Komoniko, a site connected to a central town). All sites surveyed were artisanal gold mines. This paper draws primarily from a series of 45 in-depth interviews with women in ASM households, with additional contextual information from a larger cross-sectional survey. Please see Table 1 for additional details on the mining sites surveyed.

**Table 1:** Study site characteristics.

Kouroussa				
Village Name	Distance from district capital (Commute time)	Total Population	Eligible study population (est.)	Active mining at time of study
**Djikoudoumba**	25 km	2000	320	Yes
**Komoniko**	<5 km	1500	240	Yes
**Koumana**	30 km	500	80	Yes
**Bakroukoni**	80 km	3000	480	Yes
**Sagbakoro**	75 km	500	80	Yes
**Balan**	8 km	1000	>200	Yes
Siguiri				
**Barada**	38 km	1500	240	Yes
**Nafadji**	62 km	1500	240	Yes
**Faradjè**	61.5 km	1250	200	Yes
**Doko**	16 km	1000	150	Yes
**Kolenda**	25 km	800	100	Yes
**Mankango**	71.5 km	350	56	Yes

### 2.1 Cross-sectional survey

A cross-sectional survey of households was carried out in two waves, August–September 2018 and March–April 2019, with a total sample size of 613. Households were chosen randomly, with sample size in each site proportional to the population size. Households were eligible to participate if at least one household member was active in mining and if at least one child under the age of five lived in the household. The mother or guardian of the youngest child was the primary respondent. All women interviewed cited mining as either their full- or part-time livelihood. All but two interviewed participants were both caretakers and cited mining as one of, if not their primary, livelihood. Quantitative data were entered into smartphones and uploaded to a cloud-based data storage platform, then cleaned and analyzed using Stata SE15 (StataCorp, 2017) . During preliminary data collection carried out in May 2018, the study team worked with local authorities to generate rough estimates of mining camp populations. From these lists, estimated sample sizes were generated for each participating village, based on their estimated population (see Table 1 for full details). Participants for interviews and the household cross-sectional survey were selected randomly using the following procedure. Using a local guide, the rough center point of the village was determined, with each interviewer walking in opposing directions to ensure that each quadrant of the village was covered. Interviewers were instructed to leave at least three to four households between each potential participant. While efforts were made to maintain village quadrants, villages were not always laid out in geometric patterns. In these cases, interviewers were instructed to walk in opposing directions, and to continue until reaching the edge of the village, to avoid potential overlap. Please refer to Table 1 for estimated population sizes of each surveyed village.

### 2.2 In-depth interviews

A team of three local researchers conducted in-depth interviews with a smaller subset of the study population. Before conducting interviews, the research team reviewed interview guides and agreed upon a spoken translation of the questions into Malinké. In-depth interviews were structured around six primary topics: demographic and migration data, food purchasing behaviors, household food roles and responsibilities, constraints and barriers regarding food choices, food production practices, and food preferences. While specific questions were pre-generated for each primary topic, interviewers were encouraged to ask follow-up and probing questions when appropriate.

Participants were eligible if they were the mother or female guardian of a child under the age of five, had someone in their household active in the ASM sector, and were able to give informed consent. Study team members were instructed to leave 3–4 households between each attempt before recruiting another household into the study. An early concern noted in the research planning was availability of participants who would most likely be at the mining sites during the day. To counter this, the study team would arrive at the study site early in the morning to interview participants before they left for the day, and would stay late at the site to interview individuals after returning from the mines. Because this left limited time to conduct interviews, the study team employed a large number of data collectors to achieve the desired sample size. Typical case sampling – a strategy that seeks to identify participants that represent routine examples of the phenomenon being studied (Patton and Patton, 1990) – was used to identify participants. In-depth interviews lasted on average 60–90 min. Interviews were conducted in the participants’ native language, with enumerators taking detailed notes and transcribing directly key phrases and statements. As participants were largely hesitant to be recorded during the interview, the study team chose not to record interviews. At the end of each day, the author and the team supervisor “interviewed” each data collector, asking about themes and interesting findings emerging from participant responses. This “interviewing of the interviewer” technique allowed for the research team to capture information-rich details from interviews and provided a space to immediately analyze findings (McMahon and Winch, 2018).

Field notes were organized and entered into Atlas T.I. (Atlas, 2017). A deductive coding scheme was created based on interview questions and applied to all data. From this initial process of assigning codes, similar results were grouped together into broader thematic categories. Code summaries were created and used to further organize analysis.

## 3 Results

### 3.1 Demographic information and Women's livelihood activities

A total of 45 women were interviewed, ranging from 18 to 45 years old. The number of children per participant ranged from 1 to 7 children, with over half (56%) having three children or fewer. The amount of time active in the mining sector ranged from as little as six months to as long as 20 years. The majority of women interviewed (51%) had been active in ASM for four years or less at the time of the interview. In-depth interview participants closely resembled the population of the larger cross-sectional survey in age, number of children, and engagement in mining activity^1^ (World Bank, 2019). Women fill largely supportive roles at the mining sites – pulling excavated material out of the shafts using buckets, washing and sorting removed material, and transporting material to sites for further processing or eventual sale.

Only 2.5% of participants in the cross-sectional survey cited agriculture as their primary economic activity, while 78% reported mining as their primary economic activity. Despite agriculture's minimal presence as the primary economic activity, 60% of women said that they had cultivated agricultural land within the last two years, while 15% had access to a garden in their household.

While men were not the focus of the interviews, results from interviews and cross-sectional surveys offered some insights into the type of work available to men in these communities. During the household survey, participants were asked if another household member was involved in mining, with 84% responding yes. The four most common responses were husbands (30%), fathers (36%), brother/sister (24%), or brother/sister in law (34%). Particularly for those camps located far from urban centers, few other livelihood activities are available, apart from small-scale commerce, services, or food vending – none of which offer the same potential earning opportunities as ASM. One particular population we interviewed as part of this larger study was food vendors. While the majority of these vendors were women, a few men were identified. Male food vendors were almost exclusively proprietors of small “cafeterias” that sold a limited range of food options, such as eggs and sandwiches. Table 2, below, presents demographic information from both the in-depth interviews and the larger cross-sectional survey.

**Table 2:** Demographic characteristics of in-depth interview and cross-sectional survey populations.

Demographic Characteristics	% of In-depth interview participants (45)	% of Cross-sectional survey participants (613)
Average Age (mean)	27.2 years (n = 42, 3 unknown)	26.1 years
Highest level of education received		
**None**	69% (31)	72% (441)
**Any other education (includes primary, secondary, adult, or madrassa schools)**	31% (14)	28% (172)
Number of children in household (mean)	3.3 (43)	4.6 (613)
Mining Status		
**Full time**	68% (30)	78% (reported as main economic activity)*
**Seasonal**	27% (12)	
**Unknown/Other**	5% (3)	
Prefecture		
**Kouroussa Prefecture**	56% (25)	45.35% (278)
**Siguiri Prefecture**	44% (20)	54.65% (335)

*It should be noted that for both in-depth interviews and household cross-sectional surveys, eligibility criteria included having a household member be active in the ASM sector and having a child under the age of 5. However, during the in-depth interviews, participants were asked if they were active in mining either on a full time or seasonal basis, while in the household cross-sectional survey households were asked to report their primary economic activity. This explains the difference in presentation regarding mining status.

### 3.2 Food preferences, choices, and accessibility

A variety of meat, fish, starches and some vegetables – such as eggplant, African eggplant, and okra - are available on any given day. Onions, green onions, and hot peppers are routinely purchased to add flavor to meals. All vegetables described as routinely purchased by women are grown locally, either outside the mining camps themselves, or in surrounding villages and towns that provide resources to the mining camp. Other items, such as commercially-produced food and snacks, are typically sourced from more distant locations, such as Conakry or Mali. Local food vendors were individuals who have relocated to the camp to sell to miners and take advantage of the large number of potential consumers. These individuals typically were from areas surrounding the mining encampment, although some migrated from further distances. Additional interviews conducted with food vendors demonstrated two distinct categories: women not engaged in mining who sold pre-prepared meals – usually rice and a sauce, with or without meat – and men who ran small “cafeterias” that sell “quick food items” such as omelets, sandwiches, tea, cold drinks, or commercially-produced food stuffs. Use and selection of these vegetables follows pre-determined recipes learned from older female family members.

Rice was routinely identified by participants as a key staple food item. Rice can be prepared and eaten in a variety of ways for breakfast, lunch, and dinner; is frequently identified as it is a source of comfort and familiarity; and can either be grown locally or imported. Most participants expressed the belief that a meal was not complete without rice.

“A complete meal is rice prepared with sauce^2^, with all of the ingredients (42-year-old mother of 7).

Preference for a particular starch (such as rice or maize) was largely determined by where the participant was from and what their mother had prepared when they were a child.

Primary characteristics driving individual food choices were the manner in which the food was prepared, family preference, whether or not the food was considered healthy, and price. When selecting vendors for both pre-made meals and raw ingredients, cleanliness of the vendor is assessed through visual inspection of the vendor and their products. Familiarity with a given vendor also played an important role in assessing cleanliness, as vendors with whom participants had previously interacted were preferred over unknown vendors. While participants stated a strong preference for meals prepared at home, when circumstance dictated (such as illness and time constraints), pre-made meals would be purchased.

The amount and type of food purchased largely depended on available financial resources. Successful days at the mine would be followed by purchases of larger quantities of staple food items, meat, and ingredients typically associated with special or holiday meals, such as potatoes. On less successful days, limited quantities of only the simplest ingredients would be purchased, sometimes on credit. When credit was not an option, or was not enough to feed the entire family, the participant would typically not eat that evening. Food is routinely purchased upon returning from the mining site, therefore what is prepared for the evening meal is regularly a reflection of a miner's daily success. One example of the mine's influence can be seen in the decision to prepare a meal called *lafidi*, which is routinely prepared when resources are limited. *Lafidi* is a simple dish made with rice, red palm oil, okra, and a seasoning called *soumbara* (described as a locally produced bouillon cube).

“I make *lafidi*, because that money [approximately $3 USD] is not enough to buy rice and make sauce. But, with *lafidi,* it is only a few ingredients, such as red oil, okra, soumbara, Maggi cube, and a little bit of hot pepper, if you want.” (35-year-old mother of 6).

### 3.3 Women's domestic workload

While the majority of participants stated that the husband is the normal food purchaser (62.5%), in practice this responsibility is often split between women and men, with women responsible for purchasing limited quantities of food, enough for the day's meal, while men are responsible for bulk purchases of rice and for providing money to purchase food. However, women also frequently reported purchasing food with the money they earned themselves from working at the mine. Approximately 80% of women reported being able to independently purchase small amounts of cheap or inexpensive foods. This is highlighted in women's responses on daily food habits:

“because it is his responsibility to buy rice for his family, and it is my responsibility to cook and to buy ingredients.^3^” () (23-year-old mother of 4).

The time demands of mining make other household tasks difficult to accomplish. Women will rise early in the morning to prepare leftovers for the morning meal, before arriving at the mining site by 8:00 or 9:00 a.m. Women are encouraged to work as much as possible, as payment for their labor is contingent on finding gold. Gold that is found over the course of the day is then sold to intermediary gold traders, who provide payment to miners, typically on a daily basis or whenever gold is found. This system also encourages spending as many days as possible at the mine – often 5–6 days per week (with the mine normally being closed one day a week). The link between time spent in the mine and the increased probability of finding gold exerts significant pressures on women's limited time.

Female miners return late, often not until 6:00 or 7:00 p.m. This means that they and their children eat late, as a full day at the mine does not release a woman from the responsibility of preparing her family's evening meal.

“Mining affects housework because if I am not at the house and I'm at the mining site, my children have difficulty finding food.” (28-year-old mother of 4).

Preparing and cooking meals remains a female responsibility, even when both men and women are engaged full-time in strenuous mining work. Different tasks are assigned to women and girls, depending on their age, household status, and the presence of other female household members. Young girls are delegated fetching and cleaning tasks, such as retrieving water from a pump or well and washing dishes. Once a girl is old enough, her mother or another female relative will teach her how to prepare different meals, with a greater share of responsibility moving to older daughters over time.

“It is my daughters that help me cook, myself now I don't cook, I taught them how to so that I could rest, now that she is older, she can cook.” (Age unknown, mother of 6).

If no daughters are old enough, participants seek assistance from other adult female family members, such as co-wives, female siblings, and rarely mothers-in-law. In some households, a system of task-sharing between female household members was created to help balance time demands in the mine with household responsibilities.

“Because here I am the only woman, there are many of us in the house, if it is your turn you take care of it yourself, at this time the other women are in the mines working.” (36- year-old mother of 2).

Cooking is a female responsibility, and many participants expressed strong views opposing the idea of a male cooking for his family. Participant responses ranged from pity for the husband who does not like what his wife cooks, to judgment of a person's character as a spouse, to expressing that a man cooking would be a taboo act.

“A man who cooks a meal for his family is a man who is *molakonya [dishonest or undignified]*, a crazy man.” (42-year-old mother of 7).

“I think that when a man cooks for his family this is not a good thing because men are not made for cooking.” (30-year-old mother of 4).

“I think that a woman who does not cook for her family is someone of poor character.” (45-year-old mother of 3).

Other responses underlined a realistic pragmatism on the part the participants, who acknowledged that if the wife is sick or otherwise absent, the husband might have to cook for his family. However, alternative strategies were offered that did not involve the man cooking – rather, they would purchase a pre-made meal for themselves and their children.

“If I am absent, my husband buys food, I know that a lot of men do this.” (23-year-old mother of 3).

### 3.4 Mining and income instability

For the study population, mining represents their primary economic activity. While some mines close during the rainy season, approximately two-thirds of participants mine year-round, with almost four out of five participants stating that they mine five to six days per week. All women interviewed worked in supportive roles at the mining sites – i.e. washing, transporting, processing, and removal of materials from the mining shafts.

Participants gave mixed responses when explaining mining's impact on their lives. The move to ASM had largely allowed participants to meet their basic needs for food and shelter.

“Mineral extraction has changed my life a little bit, because I can buy, today, all that I need without having to ask for help from someone else.” (30-year-old mother of 4).
“Before mining I worked in the fields, but since I started mining, I have been able to meet my basic needs.” (Age unknown, mother of 6)

The ability to meet their family's basic needs through their own work was a source of considerable pride for participants. In addition to meeting basic needs, many participants were able to address additional priorities, such as constructing homes, paying for school fees, purchasing material goods, and supporting other members of their family, including elderly parents and younger siblings.

“Since I started mining, much has changed, because if I get money from mining, I can buy domestic animals and raise them, clothes, I can help my husband with purchases; I have been able to set up my house very well, and when I leave I can look very put together in front of others.” (23-year-old mother of 4).

“Since we started mining, we have worked a lot, because we have built a house and we don't have to ask anyone for our basic needs.” (25-year-old mother of 4).

For many of the interviewed women, mining represents a greater opportunity than agriculture to potentially earn more income that would provide for their families. Many participants stated that they had started mining because their friends and families had told them of the opportunity to earn additional income. The majority of interviewed women shared similar versions of the story of coming to the mines to earn a higher income than what they had been earning previously.

Despite the benefits, participants noted that the lack of guaranteed payment at the end of a day's work was a significant challenge.

“… sometimes you can spend the entire day without finding anything, and if you don't find anything, you can't eat.” (36-year-old mother of 2).

“I can only eat based on what I earn, and it isn't every day that I find gold; sometimes you can spend the entire day working and not earn anything, and so you are forced to take out credit from the vendor.” (35-year-old mother of 6).

Miners in a given group earn an income based on what is pulled out of the shaft. Even while working as a family unit, if a particular shaft is unproductive, none of the family members will be able to sell their findings to earn a daily income.

Quantitative data confirmed considerable income variability among the survey respondents. In an average week, median earnings reported by miners were approximately $10 USD. However, responses varied significantly across respondents. The standard deviation of earnings reported for an average week was approximately $10 USD, demonstrating a wide range of what is considered average by participants. Table 3 presents the income data reported in the cross-sectional survey.

**Table 3:** Reported earned income, artisanal gold miners in Northern Guinea.

	*Mean*	*Median*	*n*
Average week USD	11.63	9.79	435
Very good week USD	40.02	27.20	264

This uncertainty surrounding the anticipated income a female miner can earn in a day significantly impacts food purchasing capacity. As “time is money” in mining, miners feel pressure to spend as much time as possible working, leaving less time for cultivating other sources of food or engaging in other commercial activities. However, most women see no other realistic options to make a living.

“Mining is not good, it is not a good profession, I am only here with these difficulties because there is nothing else to do.” (28-year-old mother of 4).

The nature of women's responsibilities at the mines also contributes to their overall financial uncertainty. Shafts, or holes, are dug vertically using hand tools, with material (a mixture of ore and rock) removed by hand with buckets. This mixture then requires further processing through hand-pounding, washing, or through separation using a small hand-operated machine. Sites are typically managed by groups of individuals who have chosen to work together. A typical payment sharing arrangement identified in the surveyed mining sites between male diggers and female workers is that the woman will pull up, transport, and wash a pre-specified number of loads, with any gold in those buckets belonging to the digger (minus a small share for the owner of the land or equipment used). The next load's contents will then belong to the woman, and the count will begin again. The result is that an average woman makes considerably less than an average man—though also faces less danger, as she is not underground exposed to higher risks of shaft collapse.

While women tend to earn more (based on self-reporting by the interview participants) mining than they would otherwise earn from other livelihood activities such as farming, the varying and unpredictable nature of their payments render routine purchases, such as daily food items, difficult to cover. While husbands are often listed as the primary responsible party for food purchasing, they are often restricted by the same conditions surrounding income variability as their wives: a poor earnings day by the woman can thus not necessarily be compensated by the earnings of her husband. Saving earnings from a particularly successful day at the mine could be an effective means of gaining stability, however, as stated earlier by women when describing their purchasing habits, higher earnings on a given day typically translates to purchases of larger quantities of staples or meats and other items reserved for special occasions. Additionally, the lack of access to refrigeration precludes the storage of most items, apart from grains and select other foodstuffs.

## 4 Discussion

Informal mining communities are active, established communities that, on the whole, provide more opportunities to earn an income than do traditional farming livelihoods. Despite this increased earning potential, participants still face significant financial, temporal, and gender pressures influencing their food decision-making. The benefits of increased income earning opportunities in these informal mining communities come with increased pressures on women's time; impacting other traditional gendered responsibilities.

### 4.1 Income instability and food choices

While female miners' income is variable, their daily purchasing needs are constant, and relatively few options are available to cope with this misalignment. Food choices are influenced through a variety of factors, such as resources, convenience, and social frameworks in which individuals exist (Furst et al., 1996; Sobal and Bisogni, 2009). In the study context presented here, available resources and convenience play significant roles in a woman's food decision-making. In studies across a variety of rural, low-income contexts, one of the primary drivers of food security is income, or more frequently, the lack of income (Tangtrakul, 2010; Bashir and Schilizzi, 2013; Gebremichael and Asfaw, 2019). In a meta-analysis of 40 studies conducted in SSA and South East Asia, Bashir and Schilizzi observed that one of the strongest predictors of household food security was monthly household income (Bashir and Schilizzi, 2013). They also noted that while household monthly income was an important predictor, the distribution of income within the household was a key nuance in understanding the household's food situation. Previous literature has demonstrated the important role that women play in ensuring sufficient household food security (Hyder et al., 2005). When women in this context had the available resources, they are able to purchase sufficient and varied types of food for their families. The lack of these resources, however, pushes them to spend more time in the mines to earn an income, leaving less time to focus on food acquisition and preparation, or pushes them to adopt potentially harmful coping strategies, such as purchasing limited and basic foodstuffs on credit, or skipping meals themselves to ensure enough food for their families.

### 4.2 Mining, Women's workload, and food choice

Women must also navigate food decision-making within the social and gendered contexts of the ASM camps. While women make up approximately half of the ASM workforce in SSA (Guédé et al., 2019; Huggins et al., 2017), their contributions are often unrecognized, under-valued, or viewed as supportive of the main labor mining provided by men (Guédé et al., 2019; Huggins et al., 2017; Buss et al., 2019). Women assume a “double-burden” of social and reproductive responsibilities in the home as well as an expectation to contribute to mining production (Buss et al., 2019). Women are also expected to be more flexible in their time commitments and, when needed, take on additional household and agricultural responsibilities (Reichel, 2019). Within this context of undervalued, undercompensated, and supportive work, women must also navigate complex food choices and decision-making processes.

Within personal food systems, individuals must also negotiate and balance food values they have developed over time and through personal experiences and eventually form routines and strategies (Sobal and Bisogni, 2009). “Value negotiations”, in which an individual considers elements such as quality, relationships, monetary considerations, convenience, and health and nutrition, also play a significant role in food decision-making. Women, faced with significant time demands, must often negotiate a full day of work mining with the remainder of their household responsibilities – a major component of which is the acquisition and preparation of food for the household (Furst et al., 1996). Because of these time constraints, women rarely purchased more than what was needed for the evening meal, and relied heavily on trusted and known vendors.

While a majority of participants cited that it is the husband's responsibility to purchase food for his family, in practice this responsibility is shared between men and women. Women frequently make small daily purchases, including rice, oil, vegetables, and spices to supplement and top up larger purchases made by their husbands. Women and their husbands typically work together to purchase food, combining their resources as necessary to meet household needs. However, women frequently cited lack of resources as a barrier to accessing food, suggesting that even with multiple miners in a household, lack of financial resources can often prevent women from fully accessing all available food in their communities. Previous studies have shown that the overwhelming burden of food acquisition and preparation falls to women (Hyder et al., 2005). Women are faced with the increased challenge of not only needing to fill in the gaps in their family's daily food consumption but also to do so within an economic structure that devalues and unequally compensates their labor.

### 4.3 Mining, agriculture, and food choice

Traditionally, mining has been viewed as a direct competitor with agriculture and as a livelihood that threatens traditional agricultural through the destruction of arable land and the drawing away of laborers from farming activities (Ofosu et al., 2020). A qualitative survey of an ASM camp in rural Upper East Region, Ghana found that women there reported a lower frequency of fruit and vegetable consumption than did otherwise similarly matched women in the region, as well as higher rates of consumption of sugars and fats (Long et al., 2015b).

A growing body of literature, however, has examined the dynamic relationship between mining and agriculture as one that is supportive and cooperative (Hilson and Garforth, 2012; Cartier and Burge, 2011; Ofosu et al., 2020; Maconachie and Binns, 2007; Banchirigah and Hilson, 2010). Several authors have examined what they call “demand-pull” and “distress-push” factors (Hilson and Garforth, 2012; Banchirigah and Hilson, 2010). Distress-push factors push households into mining for lack of any other alternatives because of the unsustainability of farming. Demand-pull factors see mining as a conscious choice by households to branch out and begin mining as a way to improve their financial situation. In both settings, mining and agriculture complement each other to support the household. These activities are often seasonal, with different activities predominating throughout the year.

Mining can offer additional resources that influence food choices made by women and fill household food gaps. In the surveyed camps, however, mining has largely replaced agriculture as the primary full-time economic activity – mining “guarantees life” and allows participants to meet daily needs (Cartier and Burge, 2011). Most participants mine full-time year-round – less than half reported engaging in agricultural work in the previous year. Many of the participants interviewed in this study reported only starting mining recently, within the last four years, and many reported moving because of marriage or because their husbands’ work. In the face of significant changes in livelihoods, environment, and social setting, familiar and comfortable reminders of where a participant is from (such as a traditional meal) take on increased importance. When navigating these transitions, individuals must reevaluate their values and ideals, renegotiate relationships, and reprioritize decisions about household food intake. Food choices also differ across different types of mining camps, as discussed in a separate analysis conducted by this study on food markets that highlights three types of markets that serve artisanal miners: those closest to mining sites that offer fewer fresh fruit and vegetable options with higher proportions of prepared food options; markets on the edge of large, permanent villages located closer to mining sites; and those markets located in the center of villages or towns, with the largest proportions of raw ingredients (Zhang et al., 2020).

## 5 Conclusion

The results presented in this paper add to the ongoing discussion on the continued growth of the ASM sector in sub-Saharan Africa. In particular, we focused on food choices and how they are influenced by livelihood dynamics and gender issues. Results show that food choices in ASM camps are influenced by several factors, such as increased time demands on women through both mining and domestic responsibilities and variability in daily income. These pressures then lead women to make food choices based on what is easiest to access, what is familiar and comfortable to make in a context of multiple competing time pressures, and what is affordable.

This research was largely exploratory in nature, and further work is needed to better understand how the different drivers of food choice presented in this paper may impact household food security and nutrition status. Future research questions should focus on the impact that earning money through mining has on gender equality and women's empowerment in the camps and whether women do have more freedom to choose; whether or not the more regular (though still variable) income from mining might contribute to increased consumption of energy-dense foods that contribute to over-nutrition; and comparative studies of farmers and miners in the same area to better understand the relationship between the two, specifically as related to how food choices are made and the implications for nutrition. Future research would be well served to engage with farmers and other agriculturalists within the same geographic context, in order to gain a more complete picture of the relationship between farming and mining within these communities.

### 5.1 Limitations and strengths

Several limitations should be considered when evaluating this study. Interviews were conducted in the local language, Malinké, which the author does not speak. Field notes written in French were then further translated to English. While every effort has been made to preserve local language terms, present them in their original form, and explain their full contextual meaning, it is possible that certain cultural nuances will have failed to be conveyed accurately. Gaining consent to audio-record interviews presented a challenge. Despite the informed consent process and its emphasis on confidentiality and privacy, numerous participants expressed concern that the recordings could be used against them at a later date for taxation or legal purposes. Because of this general concern, the majority of interviews were not audio recorded. Data on income should also be viewed with a certain level of skepticism: over 20% of respondents opted not to respond to the question or claimed they did not know, likely due to hesitance to share such personal data with strangers. Collecting accurate data on earned income is notoriously difficult, particularly within the ASM context. Many of the participants mine informally and, with the presence of large multinational mining companies operating in Guinea, there exist many concerns about taxation, competition, and other interference in activities. We are thus uncertain that we are truly capturing accurate levels of household income in these sites.

There were also several strengths to the conducted research. Strong collaboration with local partners was integrated throughout the duration of fieldwork. Program timelines also allowed for extensive training and pre-testing to be carried out, helping build professional relationships among the study team and ensure efficient and high-quality data collection. Data were collected at multiple time points throughout the year to account for seasonal patterns in labor, mining engagement, and other characteristics. This allowed for a more complete description of the setting and behaviors. In addition, the use of different methods, including the collection of both qualitative and quantitative data, helped to strengthen and triangulate the results.

## Author contributions

Ronald Stokes-Walters, Mohammed Lamine Fofana, Joseph Lamilé Songbono, Alpha Oumar Barry, Sadio Diallo, Stella Nordhagen, Laetitia X. Zhang, Rolf D. Klemm, and Peter J. Winch. Conceptualization: P.J.W., S.N.N., R.D.K., and M.L.F. conceptualized the study and submitted the proposal to the Donor. Methodology: S.N.N., P.J.W., and R.D.K. developed the methodology and protocol for submission to ethical review; A.O.B., S.D., J.L.S., and R.S.W. recruited, trained, and supervised the data collection team, and ensured data quality in the field; A.O.B., S.D., J.L.S., P.J.W., and R.S.W. designed and pre-tested data collection instruments in the field; Formal Analysis: R.S.W., P.J.W., and S.N.N conducted the formal analysis presented in this paper. Writing: R.S.W. wrote the original draft of this paper; P.J.W., S.N.N., and L.X.Z. revised, provided critical feedback, and edited multiple versions of the paper. Project Administration: S.N.N., R.D.K., and M.L.F. were responsible for project administration for this research.

## Funding acquisition

M.L.F., R.D.K., and P.J.W. were responsible for acquiring funding for this research.

## Ethical approval

This research was approved by the Comité National d’Ethique pour la Recherche en Santé (CNERS) of the government of the Republic of Guinea (N: 080/CNERS/18) All participants provided informed consent prior to being interviewed.

## Source of funding and role of funder

This research was funded by the Drivers of Food Choice Competitive Grants Program, funded by the UK government's 10.13039/501100002992Department for International Development and the 10.13039/100000865Bill and Melinda Gates Foundation and managed by the Arnold School of Public Health at the 10.13039/100008899University of South Carolina. The views expressed do not necessarily reflect the UK Government's official policies.

## Declaration of competing interest

The authors declare no conflict of interest.

## Footnotes

1 As part of the larger Drivers of Food Choice Study, informal food vendors were also interviewed at the mining sites. These vendors were primarily women, most of whom had been drawn to the mining sites by the promise of earning additional income due to the increased population drawn to the mining sites. Overwhelmingly, these women food vendors did not engage in mining, instead focusing most, if not all, of their efforts on selling pre-made meals and other foodstuffs to other miners. A typical response from these women was that they did not perceive themselves as having enough time to devote to mining to make a similar income as what they earned selling food.

2 Sauce refers to whatever accompanies the starch being served with the meal – typically rice, but it could be maize or cassava as well. Ingredients are the smaller components that make up the sauce – oil, spices, etc.

3 Ingredients refer to the various food items that go into a prepared sauce.
